# Supplementary material for: Splice-Junction-Based Mapping of Alternative Isoforms in the Human Proteome
Source: Cell Rep. Author manuscript; Available in PMC 2020 Jan 15. (PMC6961840; doi:10.1016/j.celrep.2019.11.026)
Supplement: 2 [file NIHMS1546469-supplement-2.zip › DF1/PXD009737/TestisTrypsin/TestisTrypsin_58_AMHR2_PQGPAAIMETAVHHPSNWPMR.pdf]

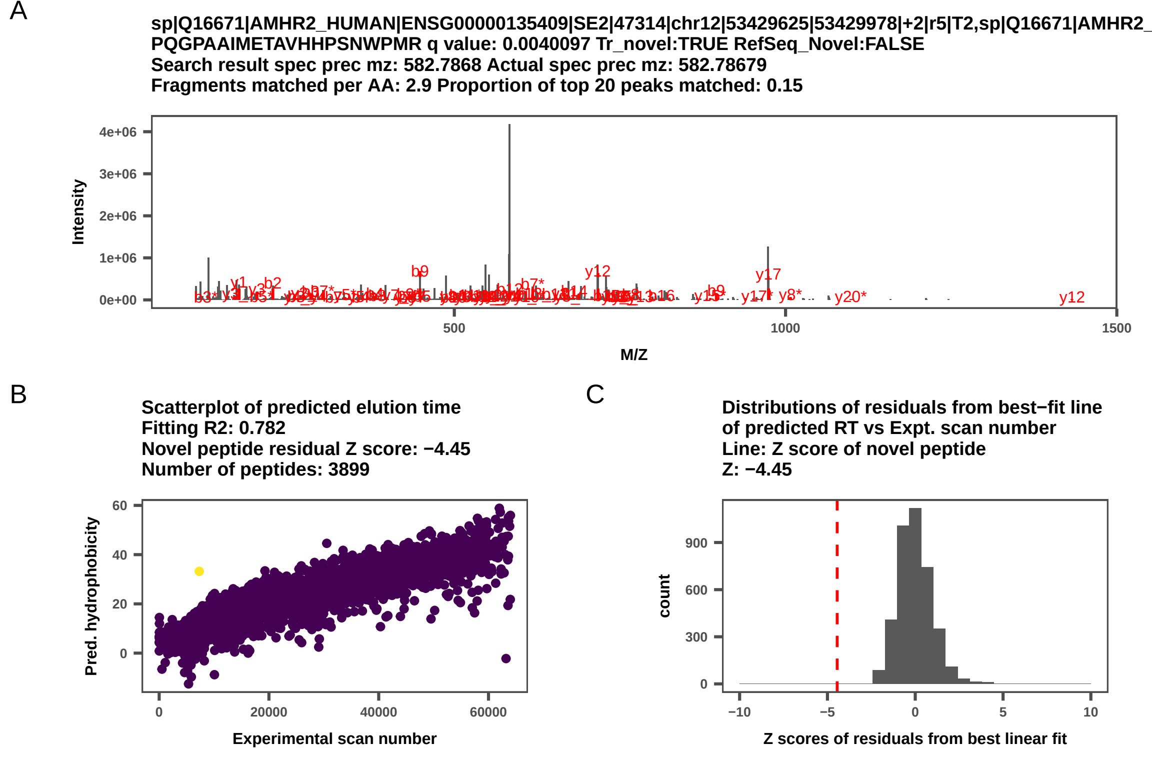
A
sp|Q16671|AMHR2_HUMAN|ENSG00000135409|SE2|47314|chr12|53429625|53429978|+2|r5|T2,sp|Q16671|AMHR2_
PQGPAAIMETAVHHPSNWPMR q value: 0.0040097 Tr_novel:TRUE RefSeq_Novel:FALSE
Search result spec prec mz: 582.7868 Actual spec prec mz: 582.78679
Fragments matched per AA: 2.9 Proportion of top 20 peaks matched: 0.15
Intensity
4e+06
3e+06
2e+06
1e+06
0e+00
b9
y12
y17
y1
b2
y20*
y12
500
1000
1500
M/Z
B
Scatterplot of predicted elution time
Fitting R2: 0.782
Novel peptide residual Z score: −4.45
Number of peptides: 3899
Pred. hydrophobicity
60
40
20
0
0
20000
40000
60000
Experimental scan number
C
Distributions of residuals from best−fit line
of predicted RT vs Expt. scan number
Line: Z score of novel peptide
Z: −4.45
count
900
600
300
0
−10
−5
0
5
10
Z scores of residuals from best linear fit
